# What Messages are Adolescent Voluntary Medical Male Circumcision (VMMC) Clients Getting and How? Findings From an Observational Study in Tanzania

**DOI:** 10.1007/s10461-016-1515-6

**Published:** 2016-08-24

**Authors:** Dorica Boyee, Erin Peacock, Marya Plotkin, Augustino Hellar, Hally Mahler, Elizabeth Edouard, Renatus Kisendi, Erick Mlanga, Emmanuel Njeuhmeli, Katherine Andrinopoulos

**Affiliations:** 1 Jhpiego, Dar Es Salaam, Tanzania; 2 Tulane School of Public Health and Tropical Medicine, New Orleans, LA USA; 3 Ministry of Health, Community Development, Gender, Elderly and Children, National AIDS Control Program, Dar Es Salaam, Tanzania; 4 USAID, Dar Es Salaam, Tanzania; 5 USAID, Washington, USA

**Keywords:** Adolescent males, Voluntary medical male circumcision, HIV prevention, Sexual health, Counseling

## Abstract

Uncircumcised adolescent males in sub-Saharan Africa are an important group to reach with voluntary medical male circumcision (VMMC) services due to high HIV burden occurring among this age group. Appropriateness of the content and delivery of sexual health and HIV prevention messages to adolescent VMMC clients has not been extensively described. A study was conducted in Tanzania to examine quality, delivery and content of messages provided to adolescent (aged 15–19) and adult (aged 20+) VMMC clients (n = 320). Results show that counseling of mixed age groups during group education lacked selected key messages, compared to more age-homogeneous groups. Additionally, adolescents received more comprehensive information in individual counseling compared to group education. We recommend that health care providers are provided with skills and job aides to assist them to segment VMMC clients by age; provide age-appropriate messages; and increase use of individual counseling as a means to communicate with adolescent clients.

**Electronic supplementary material:**

The online version of this article (doi:10.1007/s10461-016-1515-6) contains supplementary material, which is available to authorized users.

## Background

Globally, adolescents, defined by World Health Organization (WHO) as individuals aged 10–19 years, are at high risk for HIV infection: in 2012, approximately 300,000 new HIV infections occurred among people in this age group [1]. The burden of HIV infection is particularly high in sub-Saharan Africa (SSA), where 85 % of all adolescents living with HIV reside [1]. Notable reductions in HIV prevalence among 15- to 24-year-olds have been observed across SSA [2], and in Tanzania, the prevalence has decreased from 3.5 % in 2003–2004 [3] to 2.0 % in 2011–2012 [4]. However, reported risky sexual practices among adolescent males persist, including unprotected sex, alcohol abuse, and multiple concurrent sexual relationships [4–7]. In Tanzania, men report a somewhat later sexual debut than women, a median age of 18.7 years [4]. The current HIV/AIDS Strategy in Tanzania (2013) emphasizes the need for prevention of new HIV infections, including youth as a high-risk group that requires special focus [8].

Voluntary medical male circumcision (VMMC) is among the most effective HIV prevention interventions for males. Evidence from multiple randomized controlled trials has demonstrated that male circumcision reduces the risk of female-to-male transmission of HIV among heterosexual men by approximately 60 % [9–11]. Following WHO recommendations, in 2009, the Tanzanian Ministry of Health, Community Development, Gender, Elderly and Children (MoHCDGEC) produced the National Strategy for scaling up male circumcision for HIV Prevention (2010–2015), with a goal of circumcising 2.8 million men to bring the country to 80 % male circumcision prevalence among 10- to 34-year-old men [12].

Recent evidence suggests a benefit to focusing VMMC service delivery toward adolescent males [13]. Delivering VMMC to adolescents or clients under 20 years of age has several advantages, including higher social acceptance of circumcision among males in this age group, as seen in studies in Zimbabwe [14] and Tanzania [15], fewer barriers related to sexual abstinence during the post-operative healing period, and less pressure to resume sex with regular partners [13]. At scale, circumcision for adolescent males maximizes population incidence protection by providing protection from new infections throughout the entirety of their sexually active years [13].

From mid-2009 to the end of 2014, the Tanzanian MoHCDGEC, with the assistance of implementing partners and funding from the U.S. President’s Emergency Plan for AIDS Relief (PEPFAR), reached close to a million (969,500) males aged 10 and above with VMMC services [16]. More than 75 % of these VMMC clients were boys and men 10–19 years of age [16]. In Iringa and Njombe regions, where the currently described study was conducted, 78 % of the VMMC clients were under 20 years of age [17].

HIV testing is an essential entry point to HIV prevention, care and treatment. In both SSA [1] and in Tanzania, only 20 % of males aged 15–19 have been tested and received their test results [4]. HIV Testing Services (HTS) are an integral part of Tanzania’s VMMC service delivery package. The uptake of HTS within VMMC services in Tanzania has been very high: for instance, between 2009 and 2014 in the PEPFAR-funded Maternal and Child Health Integrated Program (MCHIP)-supported service in Iringa and Njombe, HIV testing uptake was 93 % among the 272,713 clients seeking VMMC services (all age groups included); 94 % of the 81,429 boys aged 15- to 19-year-olds opted to test for HIV as part of their VMMC service [17]. Thus, HTS provided within the context of VMMC offers a rare opportunity to reach a large number of young men with HIV prevention services they do not ordinarily access.

There is limited literature about health care providers’ level of comfort in discussing sexual behavior and HIV preventive practices with adolescents in the context of VMMC [18]. Studies from other adolescent sexual and reproductive services have reported adolescents’ concerns about the quality of counseling services [18, 19]. Health care providers’ inadequate counseling skills and cultural, religiously and paternalistic affected attitudes can cause major discomfort when discussing sexual health and sexuality with adolescents [20, 21]. As such, these studies in Kenya, Tanzania and Egypt have reported adolescents’ experiences with health care providers as being judgmental, harsh, advising against a reproductive health service and giving directives rather than counseling [19–21].

In a study in South Africa, adolescents expressed a desire to be able to communicate with their parents about sex and sexual health [22]. However, a review of studies in SSA shows parents who could be a primary source of information for adolescents about sexuality and health, report some discomfort discussing with adolescents about sex, condom use and sexual behavior [23]. Findings from Swaziland, Zimbabwe and Tanzania suggest that adolescents prefer to receive information on sexual and reproductive health from health care workers, and view this information as more credible than that which they receive from their parents [18, 24–26]. This suggests an especially important role for education and counseling on sexual and reproductive health within VMMC services.

In Tanzania, VMMC services are guided by MoHCDGEC protocols and WHO guidelines [27]. The protocol includes HTS as well as counseling on sexual health, HIV prevention and VMMC specific information such as post-operative care and abstinence during the wound-healing period. This information is delivered at several points along the continuum of services:

1. Before the VMMC procedure, clients receive *group education* that focuses on VMMC, HIV prevention, and other sexual and reproductive health concerns for men. Group education is an entry point where clients are given basic information before an individual counselling session.
2. During *individual pre- and post-test* *counseling*, the client undergoes a physical examination of the penis, is screened for sexually transmitted infections (STIs), counseled on VMMC and personalized HIV risk reduction and is offered an HIV test. If a test is accepted, he is given the result. In Tanzania, HIV testing is provided on an opt-out basis.
3. Immediately following the surgical procedure, the client is given *individual post-operative counseling* on wound care, recognizing danger signs, when to return for follow-up care, the need for abstinence during the healing period, and condom promotion.
4. In a 48-h follow up visit, he receives *individual follow-up counseling* on wound care and healing, abstinence for 6 weeks following the procedure, and condom promotion.

One of the challenges reported by VMMC counselors in Tanzania is segmenting the sexual, reproductive health and HIV prevention messages to be appropriate for pre-adolescent and adolescent VMMC clients. When VMMC training, job aids, and information, education, and communication (IEC) materials were originally designed, it was assumed that the client base would primarily comprise men age 20 and above. In actuality, counselors frequently encounter younger clients and have to distill messages for younger clients (who may have had limited exposure to sexual health education and may not be sexually active) from the counseling materials designed for older clients. Counselors sometimes face mixed groups which include pre-adolescent males alongside married adult men with children in the group education for VMMC services.

In 2013, a study was conducted in Njombe and Iringa regions, where VMMC services were supported by PEPFAR through MCHIP. The purpose of the study was to examine the quality and key messages delivered in HTS within VMMC sites. This paper presents findings from this study, which will help policy makers and program implementers better understand what messages health care providers are providing to adolescent VMMC clients, and will provide suggestions for improvement.

## Methods

Findings are drawn from observations of VMMC clients receiving group education and individual counseling, as part of a larger study on HTS in VMMC services. This cross-sectional study entailed conducting 320 observations of VMMC clients aged 15 years or older in 11 VMMC sites. In this paper, we refer to males aged 15–19 years as adolescents (although 10- to 14-year-olds are included in WHO definition of adolescents, they were not included in this study sample, which only enrolled VMMC clients aged 15 and above) and to VMMC clients aged 20 years and above as adults. Comparisons are made between messages received by adolescent and adult VMMC clients, and in the delivery of content by group education or individual counseling.

### Study Setting

PEPFAR defines routine VMMC services as services available and integrated within existing health care facilities, offered consistently throughout the year. In the campaign service delivery model, VMMC services are provided in high volume at outreach sites, on consecutive days for a short time period [28].

In 2012 when the study was designed, 25 government health facilities were providing VMMC services in Iringa and Njombe regions. Those sites were categorized by volume of clients and whether they were offering routine or campaign services. From the 25 sites, 11 were selected, which represented high and low volume sites and routine and campaign sites.

Three of the study facilities were providing routine VMMC services and the remaining eight facilities offered a campaign service delivery model, in an outreach setting. Facilities were stratified into routine (n = 3), outreach high (range of 60–78 clients per day) (n = 3) and outreach low settings (7–35 clients per day) (n = 5), as previous analysis showed client load could influence the quality of counseling [29].

### Study Population

All males aged 15 years or older seeking VMMC services at study sites during the period of data collection were considered eligible to participate. At the three sites with high client volume, a systematic sample of every nth eligible client was used to recruit clients. At the eight low-volume facilities, all eligible clients were recruited to participate in the study. Because participants had different probabilities of being selected into the sample (those at low-volume facilities had 100 % chance of being selected into the sample while those at high-volume facilities had <100 % chance of being selected), a sample weight (the inverse probability of selection) was calculated and applied during the analysis, yielding estimates that more accurately describe the population of clients at the selected facilities.

In addition to the written consent for surgical procedures, which is completed before every surgery per MoHCDGEC protocol, written consent to participate in the study was obtained for clients 18 years and older; and a written guardian consent and written client assent were obtained for minors (ages 15–17). Participants received a transportation allowance (USD4–6) to facilitate return to the facility for the 48-h follow-up appointment.

### Data Collection

Data were collected from January to April 2013. The study team received 2 weeks of training in quantitative and qualitative research methods, including a practicum period. The data collection team was comprised of a research coordinator, who supervised data collection, management and storage, and provided feedback on a weekly basis, and five researchers. All researchers conducting observations were between the ages of 25 and 35. Group education observations were conducted by three female and three male observers who were experienced in conducting research in a health facility setting. Individual counseling observations were conducted by a trained female nurse, and in case she was unavailable, a male researcher with extensive experience in a health facility setting carried out the observation of the individual counselling. In one clinic, in which the VMMC services were offered in a “male only” setting by male health providers, individual counseling sessions were observed by the male researcher.

To document age composition in group counseling, an observer noted the ages of all clients in attendance, using individual VMMC client records. During individual client counseling, (including pre- and post-test, post-operative, and 48-h follow-up) as well as group education sessions, the observer was present in the room and assessed session content using a standardized checklist. Additionally, a survey containing demographics, counseling experience, knowledge and attitudes was administered to all counselors working in the study facilities at the time of the study. All tools were written in Kiswahili with English subtitles.

Data were entered into Microsoft Access version 14.0 (© Microsoft Corporation) by the data management unit of Jhpiego Tanzania in Dar es Salaam. Data were cleaned, stripped of identifiers and migrated to STATA version 12.1 (© StataCorp LP) for analysis.

This study was approved by the Tulane University Biomedical Institutional Review Board and the National Institute of Medical Research Review Board of Tanzania (IRB no: 11-245721 and NIMR/HQ/R.8a/Vol. IX/1433, respectively).

### Measures

#### Message Exposure

Observers noted delivery of key messages in group education, individual pre- and post-test counseling, postoperative counseling, and 48-h follow-up counseling. For individual counseling, exposure to a particular message across ANY of the individual counseling sessions (pre-/post-test, post-operative, or 48-h follow-up) constituted message exposure. Similarly, a summary measure of exposure to a particular message was created across ALL counseling sessions: if a client was exposed to a message in group, pre- and post-test, post-operative, OR 48-h follow-up counseling, he was considered “exposed” in this analysis.

#### Client Age and Group Age Composition

Three categories of groups were created during the analysis, based on the age composition of clients in the group. A group of clients in which at least 75 % of clients were 15–17 years old was categorized as “mostly 15–17”; those in which at least 75 % of clients were 18 and older were categorized as “mostly 18+”; and a group without a 75 % majority of either age group was categorized as “mixed.” Two groups (representing eight clients) that had a 75 % majority of 10–14 year olds were dropped from the analysis of group age composition.

#### Counselor Age and Experience

Counselor age and number of months of work experience as a VMMC provider were collected in the provider survey.

### Data Analysis

The findings we present are based on analyses of individual client counseling sessions (n = 320) and group education sessions (n = 80 groups). There were 60 counselors in total, 22 of whom provided group education in the 11 facilities. To account for clustering at the facility level, robust standard errors were used. Given that participants had different probabilities of being selected into the sample, sample weights (the inverse probability of selection) were calculated and applied in the analysis to yield more accurate population estimates.

In the group education and individual counseling analyses (Tables 3 and 5, respectively), bivariate and multivariate logistic regression models were run to determine the likelihood of client’s exposure to each key message based on their age category (younger adolescents 15–17 and adults 20+years old were compared to older adolescents 18–19 years old as the reference group). Additional independent variables in the multivariate models included service delivery model, which was specified as a three-category variable for the individual counseling analysis and a two-category variable for the group education analysis (in the group analysis, one category of service delivery model predicted message exposure perfectly for three models and therefore outreach low volume and outreach high volume sites were collapsed into one outreach category).

Group education models were adjusted for counselor’s age and work experience (models were not adjusted for counselor sex as all but one counselor was female). Individual counseling models were not adjusted for counselor’s age and experience, as a client would have seen multiple counselors across the individual pre- and post-test, postoperative, and 48-h follow-up counseling sessions. Unadjusted and adjusted odds ratios are presented for message exposure by client age.

In the group age composition analysis, differences in exposure to messages by group age composition were detected using Chi square statistics (Table 4). Because of the smaller sample size at the group level (n = 80 groups), these analyses were not adjusted for service delivery model. Weighted percentages for exposure to messages by group age composition are presented.

## Results

In total 320 VMMC clients were observed, from routine sites (n = 70, 21.8 %), high-volume outreach sites (n = 136, 42.5 %), and low-volume outreach sites (n = 114, 35.6 %) (See Table 1). Among the 320 clients observed, 241 (75.3 %) were observed in both group education and individual counselling sessions; the remaining did not attend group sessions but proceeded directly to individual counseling.

**Table 1:** Overview of participants in the study

Service Delivery Model of study sites	Clients observed in group education						Clients observed in individual counseling						Total observed clients	
	Young Adolescent (15–17 years.)		Older adolescent (18–19 years.)		Adult (20 + years.)		Young Adolescent (15–17 years.)		Older adolescent (18–19 years.)		Adult (20 + years.)			
	Weighted %	n	Weighted %	n	Weighted %	n	Weighted %	n	Weighted %	n	Weighted %	n	Weighted %	n
Routine	15.7	7	30.0	14	54.3	26	13.9	9	24.4	18	61.6	43	21.8	70
Outreach low	53.6	37	12.8	9	33.7	23	29.3	37	7.0	9	63.7	68	35.6	114
Outreach high	16.0	23	46.8	53	37.2	49	16.9	26	46.9	58	36.2	52	42.5	136
Total	23.1	67	38.0	76	39.0	98	19.7	72	32.4	85	47.9	163	100	320

### Group Composition for Group Education Sessions

The 241 clients were distributed among 80 groups for group education. The median number of clients per group was 6 (range 3–27) (data not shown). Although, in many cases, the ages of clients appear to have been internally consistent (i.e. 75 % of the group was either older or younger), mixed groups were not uncommon: 26 out of the 80 groups qualified as “mixed.” Mixed groups tended to be largely composed of older adolescents (18–19 years) (42.5 %) and slightly less frequently by younger adolescents (15–17 years) (34.5 %); p < 0.001 (Table 2).

**Table 2:** Age composition of groups in group education

Participants in group counseling by age	Mostly 15–17 year olds Group (n = 10)		Mostly 18+ Group (n = 44)		Mixed ages Group (n = 26)		Total Group (n = 80)	
	Weighted % (95 % CI)	n	Weighted % (95 % CI)	n	Weighted % (95 %CI)	n	Weighted % (95 % CI)	n
15–17	76.4 (46.6–100)	21	6.1 (0.8–11.4)	9	34.6 (3.0–66.2)	34	23.5 (6.9–40.0)	64
18–19	20.5 (0–55.0)	5	36.7 (25.2–48.2)	43	42.5 (15.0–69.9)	23	37.1 (23.2–50.9)	71
20+	3.1 (0–10.7)	1	57.2 (42.2–72.1)	75	22.9 (7.2–38.7)	16	39.4 (21.3–57.6)	92

χ^2^ = 73.90, *p* < 0.001

### Message Exposure During Group education

Table 3 presents odds ratios for delivery of specific messages in group education by client age. Differences were seen in the provision of some messages according to age. Relative to 18- to 19-year-olds, younger adolescents were 3.8 times as likely to be exposed to messages about reducing sexual partners (p < 0.05), 3.3 times as likely to be exposed to messages that male circumcision does not prevent an HIV-positive man from passing the virus to his partner (p < 0.01), and 6.2 times as likely to be exposed to messages about the importance of six-week post-operative abstinence period (p < 0.05).

**Table 3:** Odds of delivery of key messages to younger adolescents and adults, relative to older adolescents in group education (n = 241)

Message	15–17 (younger adolescents)		20 + (adults)	
	Unadjusted OR (95 % CI) t, *p* value	Adjusted OR^∇,^ (95 % CI)	Unadjusted OR (95 % CI)	Adjusted OR^∇,^ (95 % CI)
Explained that the test is confidential, meaning they will not tell anyone else client results	5.0* (1.0–25.2) **t** **=** **2.24, p** **=** **0.049**	3.7 (0.5**–**26.0) t = 1.49, p = 0.168	1.0 (0.7**–**1.5) t = 0.17, p = 0.868	1.0 (0.6**–**1.6) t = −0.08, p = 0.939
Explained that the test will be performed unless client declined (test is optional)	1.0(0.2**–**4.3) t = 0.03, p = 0.980	0.9 (0.2**–**4.2) t = −0.14, p = 0.892	1.1 (0.5–2.5) t = 0.36, p = 0.723	1.7 (0.7–4.2) t = 1.40, p = 0.192
Prevention: Abstinence	3.3^ (0.8–13.9) t = 1.88, p = 0.089	3.1^ (0.8–12.6) t = 1.83, p = 0.097	1.0 (0.6–1.7) t = −0.07, p = 0.942	0.9 (0.6–1.4) t = −0.42, p = 0.684
Prevention: Being faithful to one sexual partner	5.1 (0.5–54.3) t = 1.53, p = 0.157	5.1 (0.5–53.1) t = 1.56, p = 0.150	1.7^ (0.9–3.2) t = 1.99, p = 0.074	1.8** (1.3–2.5) **t** **=** **4.18, p** **=** **0.002**
Prevention: Reducing the number of sexual partners	3.9* (1.0–14.7) **t** **=** **1.60, p** **=** **0.140**	3.8* (1.1–13.8) **t** **=** **2.32, p** **=** **0.043**	1.7 (0.8**–**3.3) t = 1.60, p = 0.140	2.2*** (1.5–3.0) **t** **=** **5.19, p** **<** **0.001**
Prevention: Wearing condoms correctly and consistently	5.2 (0.6**–**48.7) t = 1.64, p = 0.132	4.7 (0.6**–**34.7) t = 1.74, p = 0.113	2.7^ (1.0**–**7.6) t = 2.17, p = 0.055	3.6*** (2.4–5.5) **t** **=** **6.78, p** **<** **0.001**
Prevention: Condom demonstration	2.1 (0.5**–**8.7) t = 1.19, p = 0.260	2.4 (0.7**–**8.1) t = 1.64, p = 0.133	4.2** (1.7–10.5) **t** **=** **3.55, p** **=** **0.005**	5.3** (2.1–13.4) **t** **=** **3.96, p** **=** **0.003**
Prevention: Gave client condoms	3.8 (0.6**–**23.2) t = 1.64, p = 0.132	2.0(0.4**–**24.9) t = 1.14, p = 0.280	7.7^ (0.6**–**23.2) t = 1.83, p = 0.097	9.4^ (0.8**–**110.7) t = 2.03, p = 0.070
Prevention: Talk with partner about HIV prevention	0.6 (0.2**–**2.2) t = −0.90, p = 0.388	0.5 (0.1**–**2.1) t = −1.10, p = 0.298	1.9 (0.7**–**4.9) t = 1.44, p = 0.181	1.9 (0.8**–**4.8) t = 1.65, p = 0.131
Prevention: Talk with partner about HIV testing	0.5 (0.1**–**1.6) t = −1.35, p = 0.208	0.4 (0.1**–**1.5) t = −1.49, p = 0.168	1.9 (0.6**–**5.7) t = 1.30, p = 0.223	2.3^ (1.0**–**5.7) t = 2.12, p = 0.060
Explained that for HIV-positive men, MC does NOT prevent them from transmitting the virus to others	3.7** (1.7–8.0) **t** **=** **3.75, p** **=** **0.004**	3.3** (1.6–6.5) **t** **=** **3.80, p** **=** **0.003**	1.4^ (1.0**–**2.0) t = 2.21, p = 0.051	1.6* (1.1–2.4) **t** **=** **2.78, p** **=** **0.019**
Explained the importance of sexual abstinence for 6 weeks after circumcision	6.9* (1.4–33.4) **t** **=** **2.71, p** **=** **0.022**	6.2* (1.1–36.3) **t** **=** **2.32, p** **=** **0.043**	3.4** (1.7–7.0) **t** **=** **3.80, p** **=** **0.003**	4.7*** (3.1–7.0) **t** **=** **8.40, p** **<** **0.001**

Bold values are statistically significant

^ p < 0.10, * p < 0.05, ** p < 0.01, *** p < 0.001

Ref: Older adolescents (18–19 year olds)

^∇^Adjusted for service delivery model, counselor age, and number of months counselor has worked in VMMC

In group education, compared to 18- to 19-year-olds, adult men were 1.8 times as likely to be exposed to messages about being faithful to one sexual partner (p < 0.01), 2.2 times as likely to be exposed to messages about reducing the number of sexual partners (p < 0.001), 3.6 times as likely to be exposed to messages about using condoms correctly and consistently (p < 0.001), 5.3 times as likely to have been exposed to a condom demonstration (p < 0.01), 4.7 times as likely to be exposed to messages about the six-week post-operative abstinence period (p < 0.001), and 1.6 times as likely to have received an explanation that male circumcision does not prevent an HIV-positive man from passing the virus to his partner (p < 0.05).

As presented in Table 2, client age and group age composition are strongly associated, with mixed groups dominated by 18- to 19-year-olds. Interestingly (see Table 4), mixed groups were less likely to be exposed to messages about sexual behavior and sexual partnerships, including being faithful to one sexual partner (78.0 % of mixed groups received this message versus 90.3 and 100 % of mostly 18+ groups and mostly 15–17 groups, respectively; p < 0.05), reducing the number of sexual partners (73.0 % of mixed groups versus 91.9 and 100 % of mostly 18 + and mostly 15–17 groups, respectively; p < 0.01), talking with a partner about prevention (3.3 % of mixed group versus 29.6 and 14.3 % of mostly 18 + and mostly 15–17, groups respectively; p < 0.05) and talking with a partner about testing (19.8 % of mixed groups versus 59.7 and 24.8 % of mostly 18 + and mostly 15–17 groups, respectively; p < 0.05). Mixed groups were also less likely to be given condoms (2.4 % of mixed groups were given condoms versus 12.9 and 27.2 % of mostly 18 + and mostly 15–17 groups, respectively; p < 0.05).

**Table 4:** Delivery of messages in group education by group age composition

Message	Age composition				
	Mostly 15**–**17 (n = 10 groups) Weighted % (95 % CI)	Mostly 18+ (n = 44 groups) Weighted % (95 % CI)	Mixed (n = 26 groups) Weighted % (95 % CI)	χ^2^, p value	Total (n = 80 groups) Weighted % (95 % CI)
Explained that the test is confidential, meaning they will not tell anyone else client results	32.8 (0–100)	21.8 (0–46.8)	43.6 (21.1–66.1)	χ^2^ = 3.82, p = 0.467	30.6 (17.3–43.9)
Explained that the test will be performed unless client declined (test is optional)^	53.6 (13.3–93.9)	75.9 (49.2–100)	47.7 (36.1–59.3)	χ^2^ = 6.27, p = 0.069	63.7 (45.4–82.0)
Prevention: Abstinence	100	79.0 (62.3–95.8)	73.0 (46.1–100)	χ^2^ = 2.93, p = 0.138	79.2 (61.5–96.9)
Prevention: Being faithful to one sexual partner*	100	90.3 (79.6–100)	78.0 (57.5–98.5)	χ^2^ = 3.73, p = 0.028	87.1 (75.2–99.0)
Prevention: Reducing the number of sexual partners**	100	91.9 (84.0–99.7)	73.0 (46.1–100)	χ^2^ = 6.63, p = 0.009	86.2 (74.4–98.0)
Prevention: Wearing condoms correctly and consistently^	100	100	76.9 (63.2–90.7)	χ^2^ = 13.1, p = 0.068	92.0 (87.1–96.8)
Prevention: Condom demonstration	77.7 (13.9–100)	69.7 (43.7–95.7)	47.8 (32.5–63.0)	χ^2^ = 4.42, p = 0.161	62.9 (43.4–82.5)
Prevention: Gave client condoms*	27.2 (1.6–52.8)	12.9 (3.1–22.8)	2.4 (0–8.2)	χ^2^ = 4.69, p = 0.045	10.8 (2.8–18.8)
Prevention: Talk with partner about HIV prevention*	14.3 (0–55.1)	29.6 (8.1–51.2)	3.3 (0–11.1)	χ^2^ = 7.87, p = 0.041	18.8 (5.5–32.1)
Prevention: Talk with partner about HIV testing*	24.8 (0–95.5)	59.7 (40.0–79.4)	19.8 (3.9–35.6)	χ^2^ = 12.30, p = 0.048	42.0 (26.9–57.1)
Explained that for HIV–positive men, MC does NOT prevent them from transmitting the virus	57.0 (0–100)	52.8 (28.9–76.7)	42.0 (21.0–63.1)	χ^2^ = 1.01, p = 0.551	49.5 (32.8–66.2)
Explained the importance of sexual abstinence for 6 weeks after circumcision	89.8 (50.2–100)	94.5 (86.3–100)	84.6 (65.1–100)	χ^2^ = 1.96, p = 0.519	90.6 (84.6–96.6)

^ p < 0.10, * p < 0.05, ** p < 0.01

### Message Exposure During Individual Counseling (Including Pre- and Post-test, Post-operative, and 48-h Follow-up Counseling) 

In contrast to group education, adolescents were just as or more likely than adults to be exposed to prevention messages in individual counseling (with the exception of the message related to male circumcision not preventing an HIV-positive man from transmitting the virus to others). Table 5 presents odds ratios for delivery of specific messages during individual counseling, using 18- to 19-year-olds as the reference age group. The 18- to 19-year-olds were more likely than adults to be exposed to messages related to confidentiality of HTS (OR for adults relative to 18- to 19-year-olds = 0.3, p < 0.01), abstinence (OR for adults relative to 18- to 19-year-olds = 0.6, p < 0.05), and using condoms correctly and consistently (OR for adults relative to 18- to 19-year-olds = 0.4, p < 0.01).

**Table 5:** Odds of delivery of key messages to younger adolescents and adults, relative to older adolescents in individual counseling (pre and post-operative and 48-h visit) (n = 320, unless otherwise noted))

Message	15–17 (adolescents)		20 + (adults)	
	Unadjusted OR (95 % CI)	Adjusted OR^∇^ (95 % CI)	Unadjusted OR (95 % CI)	Adjusted OR^∇^ (95 % CI)
Explained that the test is confidential, meaning they will not tell anyone else client results (n = 315)	2.2 (0.5–9.0) t = 1.23, p = 0.246	0.9 (0.5–1.6) t = −0.58, p = 0.576	0.9 (0.5–1.7) t = −0.33, p = 0.746	0.3** (0.1–**0.6)** **t** **=** **−3.56, p** **=** **0.005**
Explained that the test will be performed unless client declined (test is optional) (n = 313)	0.7 (0.2–3.4) t = −0.47, p = 0.650	0.8 (0.3–2.2) t = −0.53, p = 0.610	0.7 (0.2–2.8) t = −0.56, p = 0.588	0.8 (0.3–2.2) t = −0.49, p = 0.632
Prevention: Abstinence	2.4 (0.6–10.1) t = 1.32, p = 0.217	1.7 (0.4–7.7) t = 0.83, p = 0.428	0.8 (0.5–1.4) t = −0.84, p = 0.421	0.6* (0.4–**0.9)** **t** **=** **−2.74, p** **=** **0.021**
Prevention: Being faithful to one sexual partner	1.6 (0.5–5.2) t = 0.88, p = 0.400	1.0 (0.2–4.1) t = −0.04, p = 0.970	1.1 (0.5–2.4) t = 0.20, p = 0.848	0.6 (0.4–1.1) t = −1.78, p = 0.105
Prevention: Reducing the number of sexual partners	1.1 (0.4–2.8) t = 0.15, p = 0.881	0.6 (0.2–1.9) t = −0.94, p = 0.367	1.7 (0.6–5.0) t = 1.07, p = 0.310	1.0 (0.5–2.3) t = 0.11, p = 0.916
Prevention: Wearing condoms correctly and consistently	0.2* (0.1–**0.8)** **t** **=** **−2.69, p** **=** **0.023**	0.2** (0.1–0.5) **t** **=** **−3.53, p** **=** **0.005**	0.4 (0.2–1.2) t = −1.76, p = 0.110	0.4** (0.2–**0.6)** **t** **=** **−4.02, p** **=** **0.002**
Prevention: Condom demonstration	0.2^ (0.–1.1) t = −2.10, p = 0.063	0.1* (0–**0.8)** **t** **=** **−2.49, p** **=** **0.032**	1.2 (0.2–6.9) t = 0.29, p = 0.781	0.9 (0.2–3.4) t = −0.19, p = 0.856
Prevention: Gave client condoms	0.1** (0–**0.4)** **t** **=** **−3.88, p** **=** **0.003**	0.2** (0.1–**0.5)** **t** **=** **−4.20, p** **=** **0.002**	0.5 (0.1–2.3) t = −1.05, p = 0.320	1.0(0.5–2.2) t = 0.13, p = 0.900
Prevention: Talk with partner about HIV prevention	0.2** (0.1–0.5) **t** **=** **−3.71, p** **=** **0.004**	0.3** (0.1–**0.6)** **t** **=** **−3.70, p** **=** **0.004**	0.5 (0.2–1.3) t = −1.74, p = 0.113	0.6 (0.3–1.4) t = −1.31, p = 0.219
Prevention: Talk with partner about HIV testing	0.3* (0.1–0.9) **t** **=** **−2.33, p** **=** **0.042**	0.3* (0.1–**0.9)** **t** **=** **−2.44, p** **=** **0.035**	0.5** (0.4–**0.8)** **t** **=** **−3.94, p** **=** **0.003**	0.7 (0.4–1.4) t = −1.08, p = 0.306
Explained that for HIV-positive men, MC does NOT prevent them from transmitting the virus to others	1.1 (0.3–4.7) t = 0.15, p = 0.882	1.0 (0.3–3.1) t = −0.05, p = 0.962	4.0** (2.1–**7.7)** **t** **=** **4.84, p** **=** **0.001**	3.1* (1.4–**7.0)** **t** **=** **3.13, p** **=** **0.011**
Explained the importance of sexual abstinence for 6 weeks after circumcision	0.1* (0–**0.6)** **t** **=** **−3.05, p** **=** **0.012**	0.1* (0–**0.6)** **t** **=** **−2.89, p** **=** **0.016**	1.2 (0.2–7.8) t = 0.20, p = 0.849	1.5 (0.2–10.3) t = 0.52, p = 0.618

Bold values are statistically significant

^ p < 0.10, * p < 0.05, ** p < 0.01

Ref: Older adolescents (18–19 years)

^∇^Adjusted for service delivery model (routine vs. outreach low vs. outreach high)

## Discussion

Adolescents, compared to adults, are at disproportionate risk of HIV infection and HIV-related death [2, 30]. From 2005–2012, AIDS-related deaths among adolescents increased by close to 50 %; other age groups saw a 32 % decrease during the same period [1]. Reaching adolescents with HIV prevention and sexual health messages is of paramount importance, and yet, as described by a recent review article, many of the current approaches for reaching adolescents have been ineffective [31].

Overall, adolescents in our study received the same messages about HIV prevention and sexual health as adult VMMC clients. However, older adolescents were less likely to get the messages in group education. The specific messages adolescents were likely to receive in group education varied by age. Younger adolescents in group education were more likely than older adolescents to be exposed to messages related to reducing the number of sexual partners, the six-week post-operative abstinence period, and VMMC not preventing transmission of the virus to others. Compared to adults, the older adolescents in group education were less likely to be exposed to messages about being faithful to one sexual partner, reducing number of sexual partners, the six-week post-operative abstinence period, VMMC not preventing transmission of the virus to others, as well as a condom demonstration. However, the discrepancy in messages received by adolescents was balanced out in individual counseling, in which older adolescents were just as—or in the case of some messages—more likely to have been exposed to prevention messages, compared to adults.

It is notable that counselor training and the materials that counselors used as job aides contained the same content for all clients, meaning that the differences in message delivery may be based on the counselors’ comfort level in communicating subjects perceived as age-sensitive with individuals of different ages. Delivering messages in a group setting, particularly with mixed ages, may make it more difficult to convey some messages. Indeed, our presentation of the results by age group composition (Table 4) showed that mixed groups, in which older adolescents were more likely to be placed, were less likely to receive messages pertaining to sexual behavior, sexual partnerships, and condoms. It appears the counselors may have attempted to “make-up” for the lack of messaging in group education during individual counseling sessions.

In some VMMC service delivery settings, the opportunities to provide individual counseling may be shortened due to space or high volume of clients. In this case, the need to either group clients into similar ages for group education, or develop tools, including counseling scripts, to deliver messages about sexual behavior and condoms in mixed age groups becomes more important. In relation to provision of messages through group education, our findings suggest that there is a potential benefit to composing homogenous age groups for group education when possible.

The results of our study also suggest that group age composition may partially explain the variation in message exposure by age that was observed during group education. From the results, it appears that younger adolescent groups (median age 16.6) and adult groups (median age 22.7) were largely uniform; most group age diversity was encountered in the groups that included older adolescents (median age 18.6). This mix in ages for older adolescents is likely to contribute to lower exposure to key sexual health and HIV prevention messages during group education, since counselors struggle to tailor VMMC messages to their diversely aged audience.

Provision of comprehensive sexual education and adolescent-appropriate sexual health services have been described as the two most effective approaches to adolescent health services [31] [32]. A review of HIV risk factors for youth in South Africa, Zimbabwe, Tanzania, and Uganda suggests that youth-friendly services can result in increased use of health services by adolescents [33]. Although literature shows the need for providers to be trained on youth friendliness and improve communication skills, there is still a lack of clarity on how to operationalize “youth friendly services.” Our study indicates that for VMMC services, creating the right counseling environment in regards to mixed age groups is an area where additional support and guidance is needed.

In the Joint Strategic Action Framework to Accelerate the Scale-Up of VMMC for HIV Prevention in East and Southern Africa, WHO and the United Nations Program on HIV and AIDS (UNAIDS) recommend that services be refined so that they are age appropriate, youth friendly, and linked with other youth-focused programs [34]. In our study, adolescents received more comprehensive information during individual counseling compared to group education. Our findings, combined with these policy recommendations from WHO and UNAIDS, suggest that there may be a great deal of benefit to producing a tailored package that addresses adolescents in VMMC, with an emphasis on provision of the key messages related to sexual behavior during the individual counseling rather than the group education. An adolescent-friendly counseling package could contribute to increased adherence to post-circumcision guidelines, and provide young men with a positive interface with the health system.

Several limitation apply. First, accurately capturing and characterizing the provision of messages longitudinally across four different counseling sessions was challenging, and some limitations apply. While we acknowledge that the presence of an observer might have encouraged the counselors to put on their ‘best behavior,’ we feel that the presence of the research team for so many days at a site (up to 12 days per site) offset the bias inherent in having an observer present during group education and individual counseling.

Second, Tanzania does not have a national identification system, so proof of age is not required to access VMMC services. Because 18-year-olds are exempt from being accompanied by a guardian, it is possible that some VMMC clients reported being 18 years to avoid the parental consent requirement. If this were true, this would account for the unusually high number of 18-year-olds seen in the study. If these clients were actually younger than 18, counselors might have grouped them with younger VMMC clients for the group education. Third, although use of a single observer precluded determination of inter-rater reliability, this limitation would not necessarily lead to systematic bias based on age. Finally, the study included 11 of 25 sites selected purposefully based on the goals of the larger study, and may not be representative of all sites in Tanzania.

VMMC provides a unique opportunity to reach young men with sexual and reproductive health services, but in order to maximize the benefit, the service package should be tailored to the age and information needs of young men. We suggest the following to achieve this:

- Address counselors’ discomfort with giving messages to adolescents in the group education session by giving them skills in segmenting their clients by age, and training them to provide age-sensitive messages in individual counseling rather than group education.
- Create job aids that guide counselors in providing comprehensive messages, appropriately tailored to age and working with mixed age groups. These job aides should address both the messages and the suggestions on providing them to the different aged clients in a group setting.
- Individual counseling should be strengthened as a best-approach for providing key sexual and reproductive health messages to adolescents, given that young VMMC clients as well as counselors appeared to be more comfortable receiving and providing messages perceived as age-sensitive during individual counseling.

## Conclusion

Idele et al. posit that:

*“Generally, the low level of [HIV] testing among adolescents could partly be the reason for increased AIDS*-*related mortality among them. Adolescents who do not know that they are infected with HIV are unlikely to seek ART, and their diagnosis may be substantially delayed until they experience symptoms of advanced HIV disease, in many cases it will be too late for treatment”* [1].

VMMC services provide a golden opportunity to reach adolescent with HTS and to disseminate essential information about sexual health and HIV prevention. To maximize this opportunity, policymakers and implementers are encouraged to improve delivery of HTS, HIV prevention and sexual health messages for adolescents, as well as linkages to care for HIV-infected adolescents. One key may lie in providing health care providers with the skills and the tools to assist them to provide both the right messages and the right counseling environment for adolescent VMMC clients.

## Electronic Supplementary Material

Below is the link to the electronic supplementary material.

Supplementary material 1 (PDF 73 kb)

Supplementary material 2 (PDF 76 kb)

Supplementary material 3 (PDF 70 kb)

Supplementary material 4 (PDF 52 kb)
