# Supplementary material for: What Messages are Adolescent Voluntary Medical Male Circumcision (VMMC) Clients Getting and How? Findings From an Observational Study in Tanzania
Source: AIDS Behav. 2016 Aug 24;21(5):1383–93. doi: 10.1007/s10461-016-1515-6 (PMC5378739; doi:10.1007/s10461-016-1515-6)
Supplement: Supplementary file 1 — Supplementary material 1 (PDF 73 kb) [file 10461_2016_1515_MOESM1_ESM.pdf]

Tulane University Human Research Protection Office
Biomedical IRB Consent Script for Participation in a Research Study
Integration of HIV Counseling and Testing with Voluntary Medical Male Circumcision Services

Principal Investigator: Katherine Andrinopoulos
Study Title: Integration of HIV Counseling and Testing with Voluntary Medical Male Circumcision Services
Performance Sites: Iringa, Tanzania
Sponsor: United States Agency for International Development

Client observation and survey – adult

Introduction

You are invited to participate in a research study to look for ways to strengthen HIV testing and counseling when a man comes for circumcision, and linkages across HIV services. You are being asked to participate because you are a male circumcision client. In total, about 400 clients throughout Iringa will be asked to participate in this part of the study.

Why is this study being done?

We are conducting this research study to find ways to strengthen HIV testing and counseling when a man comes for circumcision and linkages across different types of HIV services. This requires that we understand how HIV testing and counseling is now being offered and discussed in male circumcision programs. We also need to know how effective HIV testing and counseling is when it is offered in male circumcision programs. That requires that we understand how much information men retain from the counseling session and what behaviors they intend to engage in, in the future.

What are the study procedures? What will I be asked to do?

If you agree to take part in this study, a research team member will observe the counseling services you are provided today and when you come back for your follow-up visit for male circumcision. When you come back you will also be asked to complete a survey. Survey questions will cover demographic, health status, and behavioral information about you. We will also ask you about your experiences with the services received and the information about the counseling session. The survey will be administered in a private room in the clinic after you have completed your follow-up appointment. The survey will take approximately 45 minutes. We will also look at information that is on your client card at this clinic.

What are the risks or inconveniences of the study?

A possible inconvenience may be the time it takes to complete the survey. If this is a problem, you can choose to end the observation and survey if you change your mind at any point.

We will be careful to keep your information private. However, a possible risk is that by accident someone outside the study team learns the information you share with us. We assure you that we will make every effort to keep your information private. We will not record your name or other identifying information on any data collection tools. Instead a code number will be used. We will secure these tools and all study records. We will not share any of the information you tell us with anyone else, including people who work at this clinic. We will combine the information you share with us with the information from other participants. We will only present this information in summary form. No one's name will be used in any presentation of the results from this study.

Some of the questions are personal in nature. Another possible risk is that these questions may cause you to feel embarrassed. If this happens, you can skip any question you do not want to answer.

What are the benefits of the study?

You may not directly benefit from this research; however, we hope that your participation in the study may help us understand how to improve HIV testing and counseling and other HIV services.

Will I receive payment for participation?

You will receive a small compensation for your participation. This is to help cover the cost of transportation back to the clinic for your follow-up appointment and survey. It is also to thank you for your time in the study. You will receive compensation for your travel cost and a small gift, together up to a value of TSH$6,000.

Are there costs to participate?

There are no costs to you to participate in this study.

How will my personal information be protected?

The following procedures will be used to protect the confidentiality of your data. Your name will not appear on any research records. The researchers will keep all study records locked in a secure location and the only people that will have access to them are members

of the research staff. All electronic files (e.g., database, spreadsheet, etc.) will be password protected. Any computer hosting such files will also have password protection to prevent access by unauthorized users. Only the members of the research staff will have access to the passwords. At the conclusion of this study, the researchers may publish their findings. Information will be presented in summary format and you will not be identified in any publications or presentations. Any data described in this paragraph will be maintained in accordance with the security provisions of this paragraph until destroyed by the researchers at the conclusion of the study.

You should also know that the Tulane University Human Research Protection Office and the Biomedical Institutional Review Board (IRB) may inspect study records as part of its auditing program. These reviews will only focus on the researchers and not on your responses or involvement. The IRB is a group of people who review research studies to protect the rights and welfare of research participants.

Can I stop being in the study and what are my rights?

You do not have to be in this study if you do not want to. If you agree to be in the study, but change your mind at any time , you may ask the researcher to stop the observation or survey. There are no penalties or consequences of any kind if you decide that you do not want to participate. You do not have to answer any question that you do not want to answer. The services you came here for will not be taken away or changed if you decide not to participate.

Who do I contact if I have questions about the study?

Take as much time as you like before you make a decision to participate in this study. We will be happy to answer any question you have about this study. If you have further questions about this study, want to voice concerns or complaints about the research or if you have a research-related problem, you may contact the principal investigator, Katherine Andrinopoulos (kandrino@tulane.edu). In Tanzania you can contact Mr. Renatus Kisendi (0753 063 323 or kisendik@yahoo.com). If you would like to discuss your rights as a research participant, discuss problems, concerns, and questions; obtain information; or offer input with an informed individual who is unaffiliated with the specific research, you may contact the Tulane University Human Research Protection Office (in the state of Louisiana, United States) at irbmain@tulane.edu.

Tulane University Human Research Protection Program
Biomedical IRB

Approved on:	12/14/2012
Expires on:	05/13/2013
Study number:	11-245721

<u>Written Consent:</u>

The information in this form has been explained to me. Based on this information, I have decided that I will participate in the research project described above. Its general purposes, the particulars of involvement and possible risks and inconveniences have been explained to my satisfaction. I understand that I can withdraw at any time. My signature also indicates that, if I have requested a copy of this form, one has been provided to me.

_______________________________________ ____________
Subject Date

_______________________________________ ____________
Parent/Legally Authorized Representative (if applicable) Date

_______________________________________ ____________
Person Obtaining Consent Date

I am unable to read but this consent document has been read and explained to me by ____________________ (name of reader). I volunteer to participate in this research.

_______________________________________ ____________
Subject Date

_______________________________________ ____________
Witness Date

_______________________________________ ____________
Person Obtaining Consent Date

Tulane University
Human Research Protection Program
Biomedical IRB

Approved on:	12/14/2012
Expires on:	05/13/2013
Study number:	11-245721
