# Supplementary material for: What Messages are Adolescent Voluntary Medical Male Circumcision (VMMC) Clients Getting and How? Findings From an Observational Study in Tanzania
Source: AIDS Behav. 2016 Aug 24;21(5):1383–93. doi: 10.1007/s10461-016-1515-6 (PMC5378739; doi:10.1007/s10461-016-1515-6)
Supplement: Supplementary file 3 — Supplementary material 3 (PDF 70 kb) [file 10461_2016_1515_MOESM3_ESM.pdf]

Tulane University Human Research Protection Office
IRB Assent Form for Participation in a Research Study
Integration of HIV Counseling and Testing with Voluntary Medical Male Circumcision Services

Principal Investigator: Katherine Andrinopoulos
Study Title: Integration of HIV Counseling and Testing with Voluntary Medical Male Circumcision Services
Sponsor: United States Agency for International Development

Client observation and survey – child assent

Who are we and why are we meeting with you?

We want to tell you about a research study we are doing. A research study is a way to learn information about something. We would like to find out more about how HIV services are provided. You are being asked to join the study because you have decided to get circumcised.

What will happen to me in this study?

If you want to be in this study, a research team member will observe the counseling services you are provided today and when you come back for your follow-up visit for male circumcision. When you come back you will also be asked questions about your experience. These questions will cover basic things about you like your age. You will also be asked questions about HIV and your behavior. We will also ask you your opinion about the services you received including information about the counseling session. We will ask you these questions in a private room after you have completed your follow-up appointment. This will take about 45 minutes. We will also look at information that is on your client card at this clinic.

Can anything bad happen to me?

Sometimes things happen to people in research studies that may hurt them or make them feel bad. These are called risks. The risks of this study are that you may not want to talk about some of the questions. You also might get bored with the questions and not want to spend time answering them. You do not have to answer questions you do not want to answer. We can skip questions you do not like. You can also decide to stop being in the study at any time.

We will keep your information private. We will not share anything you tell us with other people. However, it is possible that by accident someone else learns what you share with us. To keep this from happening, we will use a code instead of your name on the papers with your responses. We will also keep all study papers in a safe place that is locked.

Tulane University Human Research Protection Program
Biomedical IRB

Approved on:	12/14/2012
Expires on:	05/13/2013
Study number:	11-245721

Can anything good happen to me?

You may not directly benefit from this research. However, you may feel good about sharing information that may make services better for other people who use HIV services someday.

Will anyone know I am in the study?

We will not tell anyone outside the study team anything you share with us. The people at this clinic may know you are in this study because they see us talking, but everything you tell us will be kept private between you and the person who asks you questions. Remember, we will use a code number for your responses and will keep these papers safe and private. Only people who are on the research team will look at these responses and they will not know your name. Being in this study will not change anything about the services you get at this clinic.

Will I be given anything to take part in this study?

You will receive a small compensation for being in this study. This is to help cover the cost of transportation back to the clinic for your follow-up appointment and survey. It is also to thank you for your time in the study. You will receive compensation for your travel cost and a small gift, together up to a value of TSH$6,000.

Who can I talk to about the study?

You can ask us questions at any time. You can ask now. You can ask later. You can talk to me or you can talk to someone else at any time during the study.

If you have any questions about the study or any problems with the study you can contact the Principal Investigator Katherine Andrinopoulos by email at kandrino@tulane.edu. In Tanzania you may contact Mr. Renatus Kisendi. You can call him at 0753 063 323. You can also email him at kisendik@yahoo.com.

If you have any questions about the study but want to talk to someone who is not part of the study, you can call the Tulane University Human Research Protection Office (HRPO) at (504) 988-2665.

Tulane University
Human Research Protection Program
Biomedical IRB

Approved on:	12/14/2012
Expires on:	05/13/2013
Study number:	11-245721

What if I do not want to do this?

You don't have to be in this study if you do not want to. No one will get angry or upset if you don't want to be in this study. Just tell us. And remember, you can change your mind later if you decide you don't want to be in this study anymore.

Signature

If you understand this study and you are willing to participate, please sign below:

____
Subject Name

__________________________________ __________
Subject Signature Date

I am unable to read but this consent document has been read and explained to me by ____________________ (name of reader). I volunteer to participate in this research.

__________________________________ __________
Subject Date

__________________________________ __________
Witness Date

Signature of Investigators or Responsible Individual:

"To the best of my ability, I have explained and discussed the full contents of the study, including all of the information contained in this consent form. All questions of the research subjects and those of his/her parent(s) or legal guardian have been accurately answered."

____
Investigator/Person Obtaining Consent Name

__________________________________ __________
Signature Date

Tulane University
Human Research Protection Program
Biomedical IRB

Approved on:	12/14/2012
Expires on:	05/13/2013
Study number:	11-245721
