# Supplementary material for: What Messages are Adolescent Voluntary Medical Male Circumcision (VMMC) Clients Getting and How? Findings From an Observational Study in Tanzania
Source: AIDS Behav. 2016 Aug 24;21(5):1383–93. doi: 10.1007/s10461-016-1515-6 (PMC5378739; doi:10.1007/s10461-016-1515-6)
Supplement: Supplementary file 4 — Supplementary material 4 (PDF 52 kb) [file 10461_2016_1515_MOESM4_ESM.pdf]

Tulane University Human Research Protection Office
Biomedical IRB Consent Script for Participation in a Research Study
Integration of HIV Counseling and Testing with Voluntary Medical Male Circumcision Services

Principal Investigator: Katherine Andrinopoulos
Study Title: Integration of HIV Counseling and Testing with Voluntary Medical Male Circumcision Services
Performance Sites: Iringa, Tanzania
Sponsor: United States Agency for International Development

General provider - observation

Introduction

You are invited to participate in a research study to look for ways to support HIV testing and counseling (HTC) when a man comes for circumcision, and linkages across HIV services. You are being asked to participate because you are a provider of services to men seeking circumcision services. In total, 400 clients of male circumcision services will be observed throughout Iringa. If you agree to be part of the study today, you will finish your participation today and we will not contact you in the future.

Why is this study being done?

We are conducting this research study to find ways to support HIV testing and counseling when a man comes for circumcision, and linkages across HIV services. That requires that we understand how HIV testing and counseling is now being offered and discussed in male circumcision programs. We also need to know providers' perspectives on integrating HIV testing and counseling into male circumcision programs.

What are the study procedures? What will I be asked to do?

If you agree to take part in this study, you will be asked to allow an observer to enter the counseling room with you and observe your interaction with the client. The observer will remain quiet and take notes during the observation. You will not be asked any questions by the observer.

What are the risks or inconveniences of the study?

It is possible that you may feel uncomfortable having a person observe your interaction with the client. The observer will not ask you questions or talk during the observation. The observer will also not record any information that could identify you on study documents. Rather than record your name, the observer will use a study code. However, it is still possible that by accident someone outside the team learns information from our observation. To protect your privacy we will keep all information regarding your performance completely confidential. Our observations will not be shared

Tulane University
Human Research Protection Program
Biomedical IRB

Approved on:	12/14/2012
Expires on:	05/13/2013
Study number:	11-245721

with your supervisors and no one's name will be used in any reports from this study. All findings will be presented in such a way that no individual can be identified.
Please be assured that the observation is not an individual evaluation of your performance. That is why we will not record your name. Instead, we hope to learn more about the process of providing services overall in male circumcision programs.

What are the benefits of the study?

You may not directly benefit from this research; however, we hope that your participation in the study may help us understand how to improve HIV testing and counseling services for male circumcision clients and their providers, and linkages across HIV services.

Will I receive payment for participation?

You will not receive payment for the time you spend in this study.

Are there costs to participate?

There are no costs to you to participate in this study.

How will my personal information be protected?

The following procedures will be used to protect the confidentiality of your data. We will not collect your name or any other personal information that could identify you. Your name will not appear on any research records. The researchers will keep all study records locked in a secure location and the only people that will have access to them are members of the research staff. All electronic files (e.g., database, spreadsheet, etc.) will be password protected. Any computer hosting such files will also have password protection to prevent access by unauthorized users. Only the members of the research staff will have access to the passwords. At the conclusion of this study, the researchers may publish their findings. Information will be presented in summary format and you will not be identified in any publications or presentations. Any data described in this paragraph will be maintained in accordance with the security provisions of this paragraph until destroyed by the researchers at the conclusion of the study.

You should also know that the Tulane University Human Research Protection Office and the Biomedical Institutional Review Board (IRB) may inspect study records as part of its auditing program, but these reviews will only focus on the researchers and not on your responses or involvement. The IRB is a group of people who review research studies to protect the rights and welfare of research participants.

Tulane University Human Research Protection Program
Biomedical IRB

Approved on:	12/14/2012
Expires on:	05/13/2013
Study number:	11-245721

Can I stop being in the study and what are my rights?

You do not have to be in this study if you do not want to. If you agree to be in the study, but change your mind in the middle of the observation or survey, you may ask the observer to leave or stop the survey. There are no penalties or consequences of any kind if you decide that you do not want to participate. You do not have to answer any question that you do not want to answer.

Who do I contact if I have questions about the study?

Take as much time as you like before you make a decision to participate in this study. We will be happy to answer any question you have about this study. If you have further questions about this study, want to voice concerns or complaints about the research or if you have a research-related problem, you may contact the principal investigator, Katherine Andrinopoulos (kandrino@tulane.edu). In Tanzania you can contact Mr. Renatus Kisendi (0753 063 323 or kisendik@yahoo.com). If you would like to discuss your rights as a research participant, discuss problems, concerns, and questions; obtain information; or offer input with an informed individual who is unaffiliated with the specific research, you may contact the Tulane University Human Research Protection Office (in the state of Louisiana, United States) at irbmain@tulane.edu.

Verbal Consent:

Now that you have reviewed this form, please consider the purposes of the study, the particulars of involvement, and the possible risks and inconveniences. Remember that you can withdraw at any time. At this time, you may give verbal consent that you will participate in the research project described above. If you want a copy of this form, one can be provided for you.

Tulane University Human Research Protection Program
Biomedical IRB

Approved on:	12/14/2012
Expires on:	05/13/2013
Study number:	11-245721
